# Pain Sensitivity: An Unnatural History from 1800 to 1965

**DOI:** 10.1007/s10912-014-9283-7

**Published:** 2014-03-29

**Authors:** Joanna Bourke

**Affiliations:** Birkbeck College, University of London, Malet Street, London, WC1E 7HX UK

**Keywords:** History of pain, Sensitivity, Anglo-American societies, Anaesthetics, Analgesics, Chain of feeling

## Abstract

Who was truly capable of experiencing pain? In this article, I explore ideas about the distribution of bodily sensitivity in patients from the early nineteenth century to 1965 in Anglo-American societies. While certain patients were regarded as “truly hurting,” other patients’ distress could be disparaged or not even registered as being “real pain.” Such judgments had major effects on regimes of pain-alleviation. Indeed, it took until the late twentieth century for the routine underestimation of the sufferings of certain groups of people to be deemed scandalous. Often the categorizations were contradictory. For instance, the humble status of workers and immigrants meant that they were said to be insensitive to noxious stimuli; the profound inferiority of these same patients meant that they were especially likely to respond with “exaggerated” sensitivity. How did physicians hold such positions simultaneously? Pain-assignation claimed to be based on natural hierarchical schemas, but the great Chain of Feeling was more fluid than it seemed.

In 1896, a second-year medical student simply known as “E. M. P.” was working in a surgical-dressing room at The London Hospital. The hospital was located in an area of east London with a large immigrant population, allowing E.M.P. to ruminate on the relative sensitivities to pain of different ethnic and religious groups. His account—which was published in *The London Hospital Gazette*, an in-house journal for hospital personnel—epitomized a particularly nasty strand in British chauvinism. Implicit in E.M.P.’s narrative was the belief that not every person-in-pain suffered to the same degree. While certain patients were regarded as “truly hurting,” other patients’ distress could be disparaged or not even registered as “real pain.” Such judgments had major effects on regimes of pain-alleviation. At the end of the nineteenth century, E.M.P.’s condescension (if not outright contempt) for destitute, “foreign,” and other minority patients was not aberrant. Indeed, it took until the 1980s for the routine underestimation of the sufferings of certain groups of people to be deemed scandalous and, even today, the under-medicalisation of certain categories of patients continues to harm people-in-pain.

What did E.M.P. claim? He began by conjuring up an image of “Jews, Turks, and Heretics mingl[ing] together in one seething mass of injured and diseased humanity” waiting to get their wounds dressed. Lurking in a corner of the treatment room, the “sly stealthy eyes of a child of Israel” attracted E.M.P.’s attention “with a horrid fascination.” Turning away from this “uncanny object [sic] with a feeling akin to loathing,” E.M.P. concentrated on “a pleasanter sight”: two “fair haired little English boys… wearily, but patiently waiting their turn.” After giving the older of the two boys a coin, E.M.P. joined a surgeon who was preparing to operate on a “fine British working man… well developed—such a chest—altogether as powerful a man as you would meet in a day’s march.” After this metaphorical reference to British military prowess, E.M.P. observed that the surgeon took up his scalpel and asked the workman, “Are you ready?” According to E.M.P., the patient “cheerily” responded, “All right, sir,” then “grasping the back of a chair firmly,” the patient drew “a deep breath and remains—silent—motionless—till all is over.”

E.M.P. was impressed with this display of British pluck. It was in strong contrast to “a puny, wizened, shrivelled up little fellow of doubtful nationality” who “rock[ed] himself to and fro on the couch” and “repeatedly groan[ed]” when a dresser merely approached him carrying a strand of gauze. This “little writhing mass of humanity” whimpered that he could not “bear it” before “slink[ing] away amid the smiles of the stalwart Britons standing around” (89–90). For E.M.P., the editors of *The London Hospital Gazette*, and (I am assuming) many readers, physical and moral comportment during ordeals of physical suffering was a measuring-stick for a range of attributes, including social rank, level of civilisation, and refinement of sensibilities.

## Pain sensibilities

The editors of *The London Hospital Gazette* either agreed with E.M.P. or were oblivious to his blatant scorn for immigrant and indigenous working-class patients. Indeed, the Gazette routinely sneered at the local residents who made up the bulk of their patients, poking fun at their “quaint” expressions of pain and minimising the degree of distress they might be experiencing. Repugnance towards “outsider” peoples regularly focussed on their loathsome bodies: the problem was not so much that these patients “writhed” in pain, but that they were incapable of screwing up the courage to be brave in the face of misfortune. Innate pain sensitivity was forgivable; the failure to respond in a “correct” fashion was not. What E.M.P. disparaged was the inability of “Jews, Turks, and Heretics” to endure suffering with the reserved intrepidness of “stalwart Britons.”

Failure of willpower was portrayed as particularly despicable since many of these “outsiders” were believed to possess dulled sensibilities in the first place. Slaves, “savages,” and dark-skinned people generally were depicted as possessing a limited capacity to truly *feel*, a biological “fact” that conveniently diminished any culpability amongst their so-called superiors for any acts of abuse inflicted on them. Writing in 1811, for instance, “A Professional Planter” was determined not to let the evidence of anatomy dissuade him of his prejudices about the bodies of Black slaves. Although “the knife of the anatomist… has never been able to detect” anatomical differences between slaves and their white masters, he admitted, it was obvious that slaves possessed “less exquisite” bodies and minds. Because of their dulled sensitivities, slaves were better “able to endure, with few expressions of pain, the accidents of nature” (201). This was providential indeed, since they were subjected to so many “accidents of nature” while labouring in slave plantations.

The need to insist on the physical insensitivity of slaves did not diminish with the end of slavery. Quite the contrary, if hierarchies of labour and citizenship were to be retained, belief in the insensitivity of Black bodies was more necessary than ever. A year after Abraham Lincoln’s Emancipation Proclamation, anthropologist Karl Christoph Vogt provided a physiological justification for their continued abuse. Vogt’s *Lectures on Man* (1864) informed readers that “the Negro stands far below the white race” in terms of the “acuteness of the senses.” Admittedly, in hospitals that had sprung up during the civil war, “we see Negroes suffering from the gravest diseases cowering on their couches without taking any notice of the attending physicians.” But their wretched endurance was “certainly more from disposition than from habit or education” (188). In other words, African-Americans “cowered” in silent tenacity, not because of any enlightened custom or educated sensibility but simply because of a physiological disposition.

It was a myth that a generation of African-American physicians writing in the early years of the twentieth century both struggled to come to grips with and attempted to debunk. One of the main forums for this generation of doctors was the *Journal of the National Medical Association*, a journal dedicated to promoting African-American interests in medicine. In the 1914 edition, the Surgeon-in-Chief to St. Agnes Hospital admitted that there was a major debate about the ability of African-Americans to “endure pain” and “take anaesthetics.” As a generalisation, he was prepared to accept that

the Negro submits to pain with resignation, his sensibilities being less acute than those of a more highly-wrought nervous nature; that, as a rule, he is a favourable subject for anesthesia, provided his emotional spirit be not aroused and provided he have confidence in his advisers.

After this concession to those who believed that African-Americans had less sensitive nervous systems and were easily swayed emotionally, he went on to warn against translating these generalisations into more casual attitudes to providing pain-relief for African-American patients. “If you think,” he continued, “that, because the Negro is hardy and resistant, he will on that account always survive great risks at tremendous odds, regardless of circumstances, you will at some time be sorely surprised.” He pleaded with doctors to “look upon the colored patient surgically as upon a patient of any other race” (Royster 1914, 224).

But what was it about the non-European body that rendered it less susceptible to painful stimuli? Racial sciences placed great emphasis on the development and complexity of peoples’ brains. Since the “existence of feeling” depended on the “activity of the brain,” observed a writer signing himself “Philanthropos” in the early 1880s, it was logical that the “more perfect development of that organ,” the greater the perception of sensations such as pain. For him, the “rough proportion between sensibility and intellectual development” explained why “Savages will undergo [with] equanimity tortures which no civilized man (except perhaps under great excitement) could endure” (1883, 11). Or, as the author of *Pain and Sympathy* (1907) concluded when attempting to explain why the “savage” could “bear physical torture without shrinking”: the “higher the life, the keener is the sense of pain” (McCormick 1907, 10–11). In neurologist Silas Weir Mitchell’s famous statement of 1892, during the “process of being civilized we have won… intensified capacity to suffer.” After all,

the savage does not feel pain as we do: nor as we examine the descending scale of life do animals seem to have the acuteness of pain-sense at which we have arrived (108).

Racist beliefs were contradictory, however. On the one hand, non-European peoples could be denigrated as possessing lesser bodies: their position at the lower echelons of the great Chain of Feeling was due to their physiological insensibility. On the other hand, certain peoples could also be designated as inferior on precisely the opposite grounds: excessive sensitivity or, at the very least, exaggerated *responses* to pain. This was the reason medical student E.M.P. despised Jews and other “foreigners.” The chief targets in this discourse were Jews and southern Europeans. As an author writing in *The British Journal of Nursing* in 1906 asked, “Why does the Hebrew race manifest such feeble resistance” to pain compared to all other nations? (“A Toxic Theory of Pain” 1906, 333). Just a few years earlier, the author of the highly respectable textbook entitled *The Diagnostics of Internal Medicine* (1901) also accused the “Semitic stock, and the Celtic and Italic [sic] groups” of appearing to “possess an average greater sensibility to pain than the Teutonic and Slavonic groups” (Butler 1901, 35). Or, as essayist Louis Bertrand pontificated in *The Art of Suffering* (1936), people from the southern or eastern parts of Europe lacked the capacity to control themselves when experiencing pain. He also criticised “the Jews, an ancient race with a refined or decadent sensibility” for being “extremely sensitive to pain” (119). Explanations for their acute sensitivities lay as much in their physiological degeneracy as it did in their *moral* inferiority (or their inability to restrain their emotions).

A degenerate physiology was certainly one explanation for such peculiar sensitivity to painful stimuli, but, in addition, these groups were accused of possessing immature psyches. Irishmen and Jews “made the most noise on the operating table,” according to an author in the *British Medical Journal* in 1929. He claimed to have observed that

The Hebrew cried out through fear that if he failed to attract full attention he might miss some of the benefits of hospital care; while the Irishmen called loudly upon God and the saints, and wept and groaned because he was an emotional being to whose nature the repression of feeling, whether pleasant or painful, was foreign.

This physician denied that either group were cowards. Rather, Irish patients “lacked adequate psychological inhibitions” and Jews had “learnt the bitter lesson of persecution” so were keen to ensure that they were not overlooked (“Pain” 1929, 164). Either way, their lack of inhibition stamped them as inferior.

Whether generalising according to “race” or religion or drawing meticulous regional distinctions, ascriptions of pain-sensitivity registered fears and desires linked to cultural alliances and affinities rather than physiological facts. Nevertheless, these alleged physiological traits served as useful indicators for making broader social generalisations. Hair and eye colour, for instance, were convenient “stand-ins” for racial groups. Often, the racial references were implicit, as in a 1899 article in the *American Journal of Psychology* that concluded that male schoolchildren in Michigan who had “light eyes and hair” were “less sensitive” to pain than those with “dark eyes and hair” (Carman 1899, 396). Lurking behind such pseudo-surveys were assumptions that peoples from western and northern European “stock” were more stoical when compared to “newer” immigrants from more southern parts.

In 1959, this type of pseudoscientific research excited an almost feverish debate in the “Letters to the Editor” pages of the highly esteemed *British Medical Journal*. The question that ignited the debate was simple: could pain thresholds (that is, the point at which a person subjected to a noxious stimuli complained of pain) be correlated with eye colour? The editors started things off by reporting on a study of 403 patients whose teeth had been filled at the Melbourne University Dental School. They noted that the researchers had found that

the more blue the eyes the less [pain] reaction. As the colour went through blue-grey, green, hazel, light brown, and dark brown so the reaction to pain increased on the average.

This was no “freak coincidence,” the editors continued, speculating that patients with blue eyes were likely to come from “North European stock, traditionally a phlegmatic race,” unlike brown-eyed patients who were more likely to have descended from “more excitable Mediterranean peoples” (“Steely Eyes and Pain” 1959, 418). Physicians throughout Britain eagerly joined in the fray. A doctor from Hove (Sussex) maintained that amongst his patients there was a positive correlation not only between dark brown eyes and a low pain *threshold*, but also between this eye-colour and over-*reaction* to pain. He accused his more “Mediterranean” patients of being particularly “excitable” (Bourne 1959, 827). Yet another doctor in Hove pursed the argument, introducing an anti-semitic twist. For him, the positive association between brown eyes and excitable reactions to pain was due to the fact that “members of the Jewish race, in whom these physical features was present” were notorious for their “lowered [pain] threshold.” Bizarrely, he petitioned readers to investigate whether “red-haired Jews” also had brown eyes, implying that this might be significant in evaluating their degree of pain-sensitivity (Wauchope 1959, 1098).

The debate was not merely academic. Some physicians confessed that they chose their patients on the basis of eye colour. As one doctor admitted, when he was a medical student working in a casualty department,

I eased my burden considerably by always selecting blue-eyed and fair-haired children as my share of painful dressings. Nordic children either have a higher pain threshold than other children or greater self-control. I suspect the latter. At times it approaches serenity.

He believed that gender also exerted an influence, with the “Nordic girl” being better at bearing pain than her male counterpart (Hawksley 1959, 958). In such ways, beliefs about pain thresholds and pain responses translated directly into differential treatment. Ironically, in this case, patients who were perceived to be less sensitive (blue-eyed patients) won better treatment than those who were believed to be suffering the most.

## The gendering of sensitivities

Gender was equally dominant in debates about differential pain sensitivity. Were women the “weaker sex” or the “more stoical” one? In a letter to his friend Margaret King on 26 January 1792, poet William Cowper came out strongly on the side of female strength. King had written to him describing the “patience” with which a friend of hers had “endured the terrible operation of having her breast laid open,” that is, having undergone a mastectomy without anaesthetics. Cowper commented that such “patience” was “strong proof that your sex surpasses ours in heroic fortitude.” Indeed, there was “more true heroism in suffering his [God’s] will with meek submission” than there was in heroism “in a field of battle.” In war, there were a great many “incitements” to disregard pain: “renown and glory” being two important ones. In contrast, “no laurels are to be won by sitting patiently under the knife of a Surgeon,” so “the virtue is… of a less suspicious character, the principle of it more simple, and the practice more difficult” (Cowper 1792).

This juxtaposition of patience and heroism—the first being passive and female, in contrast to the active and masculine character of the second—was not always judged to be a cause for celebration. A century after Cowper’s letter, an article written by Annie Mary Brunless inverted their respective value. Brunless observed that, despite the fact that a man might be “a hopeless coward in bearing bodily pain” (even to the extent of being “fearfully depressed even by a toothache or headache”), he would still prove himself capable of dying “on a battlefield as few woman could have done.” Unlike Cowper, she disparaged women for being “capable only of patience,” while men possessed that higher virtue of “endurance” (1896, 605).

Brunless’s point was that the grand heroics involved in wounding and *being* wounded in combat were beyond a woman’s competency, but even she conceded that women might be better at bearing everyday afflictions (such as toothaches). As the author of *Passages from the Diary* (1834) put it, the “female sex” showed “great firmness” in “enduring a degree of physical pain, which would utterly break down the stubborn strength of man” (Warren 1834, 42). Women’s “patience” also impressed physician Edward Henry Sieveking. Writing in the 1860s, he believed in the “greater sensitiveness of the female.” Women’s “more delicate organisation” unfortunately meant that she was more likely to suffer a “greater frequency of painful affections.” However, “by way of balance,” women were “endowed with more placid and patient endurance than generally characterises the members of the ruder sex” (1867, 131–33). It was a theme that was still being repeated over 70 years later, when a survey showed that 70 % of physicians and dentists believed that women were superior to men in withstanding pain (Josey and Miller 1932, 375). Even at the end of the 1980s, a British study commissioned by the drug company that made Nurofen found that 75 % of people agreed that women were “better able to tolerate pain than men.” Interestingly, the generalisation was held to be correct by 86 % of women compared with only 64 % of men (Nurofen 1989, 1).

What explanations did these commentators give for women’s stoicism? For many, it was assumed to be a “natural” consequence of women’s subordination. In one particularly pessimistic account in 1910, women’s resilience was simply ascribed to their “long practice in suffering the blows of the male” (Twitchell 1910, 266). A more positive explanation alluded to women’s role as bearers of children. It was common to hear it said that “Nature, when she gave the woman that proud and exclusive duty [of childbearing], without doubt also gave her the means of discharging it,” as a physician writing in the *British Medical Journal* in 1949 put it. “I am sure that woman bears pain better and more patiently than man,” he concluded (Cook 1949, 781). As other historians have shown, this argument was highly racialised, with distinctions being made between the hardy “primitive” woman giving birth and her more sensitive “civilized” counterpart (Hoberman 2005, 86–95).

Was women’s stoicism innate or learned? Many commentators seemed to hold both views simultaneously. For instance, the Medical Superintendent of the Virol Pathological Research Laboratories, writing in *The British Journal of Nursing* in 1913 and 1914, initially seemed to be making an argument for socialisation as the chief mechanism by which women learnt to bear their tribulations. A woman who had been “trained to live for others,” he wrote, “will only complain when the pain is so bad as to interfere with her duties.” In contrast, a woman who had been “taught to think much of her own case, and to use words loosely” would “make a great fuss over slight pain, and describe it in inflated and incorrect language.” He spoke scornfully of women whose “vapourisings” were “a nuisance to everybody”: such women should not be rewarded with painkillers. However, he also seemed to adhere to the view that women were naturally stoical. After all, he admitted, “very many men” were also “addicted” to making a “great fuss” when experiencing pain and these men were “far more difficult to treat.” He maintained that “the natural tendency of a woman… is to unselfishness” while, “with men this often has to be acquired” (Gordon 1913, 22 and Gordon 1914, 27).

Occurring time and again throughout these debates, commentators frequently attempted to ensure that pre-existing prejudices about a particular group were upheld, even if it meant embracing contradictory arguments. Thus, commentators argued *both* that women were innately stoical (and therefore could be given less pain relief than men) and were profoundly weak (and thus liable to “hysterical” or exaggerated pains).

This seeming contradiction is illustrated in the views of Edward Henry Sieveking (the inventor of the aesthesiometer) and Francis Galton (the founder of eugenics). As we saw earlier, Sieveking believed that women were “endowed with more placid and patient endurance than generally characterises the members of the ruder sex.” This did not mean that women were superior to men in the way they comported themselves in pain. Sieveking was equally convinced that men were superior to women because the *nature* of their respective pains differed. Men’s pains had a much more definite, “local” character, while those of women were less discriminating. As a result, when treating male patients, the wise physician would direct his attention to “the seat of the lesion itself or the conducting nerves.” Physicians who approached female patients in this way would be “baffle[d]” because women’s pains were “commonly due to reflex or reflected irritation.” If a physician failed to “remember or recognise the sensitive organisation of the female nervous system,” Sieveking argued, they would find themselves dealing “blows at random, and in the dark, not always to the destruction of the malady or the benefit of the patient.” The more amorphous nature of women’s pains was exacerbated by “her proclivity to emotional influences, and the greater motality [sic] and excitability of her imagination.” Doctors needed to adopt a “roundabout way of examining all the organs” of the female body, in order to determine the “real source of any particular pain complained of” (1867, 131–33).

Sievekind was attempting to reconcile women’s commendable fortitude with their physiological inferiority and emotional unpredictability. Galton’s dilemma was different: he needed to reconcile his belief that a “delicate power of sense discrimination” was an indication of superiority (in which case, women were ranked above men) with his need to insist on the inferiority of (European) women in comparison with (European) men. In his *Inquiries into Human Faculty and its Development* (1883), Galton argued that all information about “natural events” passed “through the avenue of our senses.” As a result, “the more perceptive the senses are of difference, the larger is the field upon which our judgment and intelligence can act.” It followed that a chief “attribute of a high race” was acuteness of the senses. Of course, Galton elaborated, this did not imply that European women (noted for their exquisite sensitivity) were more advanced than European men. His reasoning was ingenious: sensitivity must not be confused with “nervous irritability.” European women possessed more “nervous irritability,” while their menfolk possessed “more delicate powers of discrimination” (19–20). Like Sieveking, he could admit to women’s acute senses while denying that their sensitivity was evidence of high rank.

Such attitudes affected the level of relief given to women in pain. Even in giving birth, certain types of women were deemed not to require analgesics. In the words of obstetrician G. Ernest Herman writing in 1901, the doctor’s job at a labour was to tie and cut the cord, press the placenta out of the vagina, and provide reassurance. He went on to say that “Among the higher classes he also acts as an anaesthetist,” implying that this was not the case for “lower” classes (13 and 16). Indeed, it was only from the 1940s that mainstream medical personnel began questioning assumptions that European, white-American, African-American, and “primitive” women experienced labour pains in different ways. Not surprisingly, perhaps, rebuttals were especially prominent within the African-American medical community. In 1966, for instance, William F. Mengert (Professor of Obstetrics and Gynaecology at the University of Illinois Medical Center in Chicago) published his thoughts on labour pains in an article in the *Journal of the National Medical Association*. As a medical student, he recalled, he had been taught by John Whitridge Williams, the founder of academic obstetrics in the United States. Williams had believed that “Negro babies at birth had soft heads and that these, therefore, molded easily through pelves that might otherwise cause trouble, for example, with a white baby whose head would not so readily alter its shape.” Nothing could be more incorrect, he discovered. Instead, the “attitude of the white doctor undoubtedly was responsible for most of this belief.” At Dallas, Mengert and his team had undertaken a clinical study in which

we decided not to interfere with any patient in labor until it became obvious to all [that] she could not give birth by herself. The end-point chosen was a 2 h arrest of labor after the membranes were ruptured [and] the cervix fully dilated and retracted behind the head.

By strictly adhering to these criteria, it “became obvious that Caucasian women also would give birth vaginally if allowed to labor.” Indeed, contrary to the belief that non-white women had flexible pelves, they found that “southern Negroes” were six to eight times more likely to suffer from contracted pelves than “northern whites” (this would have been due largely to inadequate diets) (413). The implication was clear: under the mistaken belief that African-American infants’ heads were softer and their mothers’ pelves more flexible (when, in fact, the opposite was the case), it had been routine to allow “southern Negro” women to labour unaided, while “northern white” women in labour were given assistance. When white women were also refused assistance, they proved just as capable of producing the infant as their darker-skinned counterparts. It is interesting that these researchers chose to withhold assistance to white women rather than intervening more in the birthing practices involving African-American women.

## The civilizing process

Ethnic, religious, and gender variations were important markers of sensitivity to pain, but they were not the only ones. Numerous commentators speculated about whether the civilising process itself had increased people’s sensitivity to painful stimuli. “Civilized man has of will ceased to torture,” argued neurologist Silas Weir Mitchell, but

in our process of being civilized we have won, I suspect, intensified capacity to suffer. The savage does not feel pain as we do: nor as we examine the descending scale of life do animals seem to have the acuteness of pain-sense at which we have arrived (1892, 108).

Perhaps one reason for the heightened sensitivities of “civilized man,” many speculated, was the availability of pain relief. Anaesthetics and analgesics had an effect on people’s *ability* (as well as willingness) to cope with acute afflictions. Physicians increasingly observed that, as “civilisation” progressed, their patients were less capable of bearing the afflictions of their flesh. It required them to treat people in their care differently, as a dentist writing in 1935 *British Dentistry Journal* noted. “There can be no doubt,” he admitted, that “our patients are now very different from the pre-war days; they are not so ready to bear pain, and are more frightened of being hurt.” As a consequence, the “old idea of the manipulation in the mouth almost regardless of the feelings of the patient has gone, and rightly so, for ever [sic] and we are at the dawn of a new era of sympathetic dentistry” (Roper-Hall 1935, 177–84).

Writing in the same decade, pioneering pain surgeon René Leriche fervently believed in the truth of this argument, illustrating it with an account of a young patient whose elbow joints had fused together after an injury and needed re-sectioning. The young man’s grandfather had undergone an identical operation after being wounded at the Battle of Sedan during the Franco-Prussian war in 1870. The grandfather had refused any anæsthetic “because he was afraid that the limb might be amputated while he was unconscious,” rendering him incapable of “mak[ing] any protest.” His grandson could not even contemplate making such a decision. Despite being “a brave, stout-hearted, energetic youth,” he “would not have allowed us to cut even a centimetre of his skin without administering an anæsthetic.” This was not a due to any “decline of moral fibre,” Leriche hastened to add: rather, it was a sign of a “nervous system differently developed, and more sensitive.” In other words, increased sensitivity to pain was a consequence of “the enhanced refinement of senses, which has advanced so rapidly during the century.” Of course, people in all times anxiously sought to shield themselves from discomfort, Leriche acknowledged, but “until recent times, they met with little success. They continued to suffer in silence, and, becoming more hardened to pain, they came to feel it less.” This meant, of course, that people in the twentieth century were “bound to suffer more readily” than their predecessors. “Even the slightest sensory disturbances,” he argued,

seem to have exaggerated importance. Far more than our ancestors, we try to avoid the slightest pain, however fleeting it is, because we know that we have the means of doing so. And, by this very fact, we make ourselves more readily susceptible to pain and we suffer more. Every time we fix our attention on anything, we become more conscious of it. So it is in the case of pain.

He argued that “by furnishing us with the means of so easily relieving pain,, antipyrine (also known as phenazone) and aspirin “rendered us more sensitive to it.” As he astutely pointed out, this change in the “sensory mechanism of mankind” had occurred at the level of “real physiology, for physiology means neither more nor less than the observation of what is occurring in ourselves” (1938, 56–7).

Leriche’s comment was directed at humanity en mass. But many commentators observed that there was significant variation *within* particular civilizations. We have already seen examples of this with regards ethnicity, religion, and gender. However, other markers tended to be categorised under two broad headings: first, personal characteristics and, second, traits shared by individuals grouped according to class or occupation.

In the first category, individuals possessed subtly different physiologies and personalities. Sensitivity to painful stimuli was often linked to an individual’s balance of the four humours, for instance. Well into the late nineteenth century, physicians argued that melancholics and those with phlegmatic (sluggish and fat) temperaments were not especially receptive to pain, in comparison to thin, excitable choleric people (Collier 1889, 624). As physiological models gradually gave way to more psychological ones, “temperament” was increasingly judged to be decisive. In an address at the London School of Tropical Medicine in 1908, for example, Sir William Bennett advised physicians to pay attention to their patients’ temperaments, comparing the reactions of a hospital porter with that of an officer “whose bravery on the field was beyond dispute.” The porter underwent his grave operation without anaesthetics and without a murmur of complaint, even thanking the surgeon afterwards. In contrast, the officer “howled loudly” despite the fact that his procedure involved simply trying to “bend a partially stiff joint.” Bennett claimed that “it would be ridiculous to attribute cowardice” to an officer who had conducted himself honourably in battle. His screams could only be explained by the fact that he possessed a “highly-strung nervous temperament which seems to be quite unable to control itself under pain inflicted in what is commonly called ‘cold blood’” (1908, 22 and 1908, 1). In other words, the officer’s nervous sensitivity could be over-ridden in the excitement of battle but was irrepressible in the stark setting of a hospital surgery.

For another group of thinkers, pain-sensitivity was, literally, embodied in the brain and skull. Phrenologists, for instance, speculated that the “Organ of Destructiveness” and the “Organ of Fighting” were crucial in predisposing men and women to physical stoicism. According to their head-map, the Organ of Destructiveness was located above the ear, extending backwards from about an inch and a half in front and top of the ears (Wells 1885, 154). People who possessed a large Organ of Destructiveness could not only “inflict [pain] upon others without compunction if not with positive pleasure,” but they could also “endure pain heroically” (Wells 1891, 165). They would “suffer without complaint” (Sizer and Drayton 1886, 127), and would even “submit one of [their] own limbs unflinching to the surgeon, if necessary” (Wells 1891, 166). Phrenologists also located the organ for insensitivity to pain in the Organ of Fighting, which lay “on both sides of the skull, near the organ of friendship, but somewhat lower, or behind, and a little above the ear” (Hufeland 1807, 92). This organ, also called the “organ of courage,” denoted “bodily courage, that disregard and inattention to bodily pain.” It was prominent in successful boxers and soldiers (Hufeland 1807, 92).

The second category of individuals who were categorised by their degree of sensitivity to pain—that is, those grouped according to class, occupation, or education—was even more common than humoral, temperamental, and phrenological ones. Who could doubt that there were crucial differences in pain sensitivity between nervous scholars and muscular agricultural labourers of people, the president of the British Medical Association asked in 1889? (Collier, 1889 624). The author of “Sensibility to Pain” (1900) concurred, drawing a positive correlation between acute sensitivity to pain and “excellent” intellectual abilities. The nervous system of brighter people “react[] quicker in response to the actions of the outside world upon them” (Swift, 1900 315–17). Unlike the physiologists and phrenologists, however, these commentators believed that the correlation between pain sensitivity and class (or education) was social rather than innate: the circumstances of a person’s life dictated whether he toughened-up or remained fragile.

In all these debates, acts of apportioning levels of sensitivity were profoundly prejudicial. “Outsider” groups—that is, categories of people who were different to those passing judgment—were in a “catch-22” situation: the alleged *insensitivity* of workers, immigrants, hysterics, and chronically-ill patients was proof of their humble status, yet the profound *sensitivity* of these same people was also proffered as evidence of their inferiority. In the context of class, middle-class commentators believed both that labouring men were insensate (because they possessed rudimentary nervous systems) and that they were oversensitive (because they lacked strength of will). Conversely, it was taken as obvious that the sensitivity of the educated, wealthy classes showed that they were highly ranked in the chain of civilisation; yet the insensitivity of the same people confirmed the fact that they possessed superior levels of self-control.

Such confused judgments even surfaced in clinical literature that purported to repudiate value judgments. For instance, John Finney was the first president of the American College of Surgeons. In his influential book *The Significance and Effect of Pain* (1914), he amiably claimed that

It does not always follow that because a patient bears what appears to be a great amount of pain with remarkable fortitude, that individual is more deserving of credit or shows greater self-control than the one who does not; for it is a well-established fact that pain is not felt to the same degree by all individuals alike.

However, in the same section, he made pejorative statements about people with low pain thresholds (they possessed a “yellow streak”) and insisted that patients capable of bearing pain showed “wonderful fortitude.” “Is there or can there be anything more sublime or more inspiring in its effect upon others than such an exhibition of self-control?” (14).

Such tensions could only be reconciled by distinguishing between pain *perception* and pain *reaction*. The civilised, white, professional man might be exquisitely sensitive to pain but, through acts of willpower, was capable of masking his reaction, which was the “sublime” example referred to by Finney. In contrast, the “savage,” the uneducated, and the dark-skinned might bear “a great amount of pain with remarkable fortitude” but was not necessarily “deserving of credit” because it was “a well-established fact that pain is not felt to the same degree by all individuals alike.” This also helps explain why both highly civilised people as well as degenerates and neurotics were said to be sensitive to pain: the civilized man had cultivated a sensitivity, which was under the control of a highly complex mind, while the degenerate was nothing more than a body-in-pain, out of control.

## A case-study in change: Erichsen’s textbook

So far, I have explored beliefs from the last two centuries about the pain sensitivities of individuals grouped according to ethnicity, religion, gender, alleged level of “civilization,” class, education, and temperament. However, the salience of these beliefs has changed over time, often with surprising rapidity, indicating that clinical notions of sensitivity to pain have been highly unstable. This is illustrated in the works of eminent surgeon John Eric Erichsen, whose *The Science and Art of Surgery. Being a Treatise on Surgical Injuries, Diseases, and Operations* went through ten different editions between 1853 and 1895. Notably, in the first and second editions of his classic text in 1853 and 1857, Erichsen was relatively uninterested in surgical pain, confining himself to very general statements about pain susceptibility. He was also indifferent to questions about the sensitivity of specific groups to painful stimuli although he did call attention to the “great difference in the mental fortitude of individuals” and to the fact that “in the heat of action” (that is, combat), injuries “often pass unnoticed” (1853, 79 and 1857, 88).

In the editions from the 1860s, Erichsen made two significant additions. First, this was the decade in which he first singled out women and children as being of “great nervous susceptibility.” Second, by the 1860s, he was emphasising the degree of pain, as opposed to the severity of injury, important because it represents a shift in Erichsen’s thinking, including an increased acknowledgement that emotional responses to “bad events” could have significant effects on physiological sensations. After all, this was the period in which Erichsen invented the concept of “trauma” in the sense we use it today, that is, as a psychological state as opposed to *τρaυμα* or bodily injury, as it meant in the original Greek. It was in the 1860s that Erichsen had observed, in the context of people who had experienced railway accidents, that there was not necessarily a direct and commensurate relationship between the degree of physical injury and nervous disarrangement (1866, 9). In surgery as well, he noted, there was no necessary correlation between the severity of the wound and the degree of pain complained of (1861, 94–5; 1864, 101–2; 1869, 107–8).

In 1872 (the first edition which had an extended section on anaesthetics) and again in 1877, Erichsen elaborated on the importance of “constitution” as well as sex, disparaging those with “an irritable and anxious mind. furthermore, it was no longer simply “women” who were less capable of bearing pain but “nervous and hysterical women” in particular. Furthermore, the moral condition of the patient, although briefly mentioned in earlier editions, took centre stage in the 1870s. Unlike earlier editions, the dangers of indulging in “excesses” were clearly spelt out in 1872 and 1877 with particular censor reserved for people whose habits could not be called “temperate and sober,” whose diet was not “sufficient and of good quality,” and whose minds had been “over-strained by the anxieties of business or the labours of a professional life.” Erichsen was most disparaging of the pain-coping capacities of

the poor inhabitant of a large and densely peopled town, who has from earliest childhood inhaled an impure and fetid atmosphere, whose scanty diet has consisted of the refuse of the shops, or the semi-decomposed offal of the stalls, and whose nervous system has been irritated and at the same time exhausted in the daily struggle for a precarious livelihood, or over-stimulated by habitual excesses in strong drinks, by which he has hoped to purchase temporary forgetfulness in the cares of a sordid life (1872, 3 and 136 and 1877, 5–6 and 180).

In other words, by the 1870s, Erichsen’s invocation of fears associated with “fetid” environments or disease-carrying miasma (a scientific theory that was in steep and terminal decline in this period) as well as his anxiety about the strains placed on modern middle-class men reflected commonly held fears of his times.

By the time the eighth edition was published in 1884, Erichsen had been appointed President of the Royal College of Surgeons and his devoted student, Marcus Beck, edited his textbook. Perhaps for these reasons, dramatic changes can be identified. The ability to bear surgical pain was still linked to an individual’s constitution, occupation, place of residence, and moral status (particularly the “sordid” imbibing of alcohol), but surgeons were also told that

Men who live hardy out-door lives are less sensitive to pain than those who follow occupations of an opposite kind. The skin, which is the main seat of sensibility in wounds, when hardened by exposure and work, is less sensitive than when it has been habitually protected from such influences.

This was a new passage, but its basic theme was consistent with earlier editions. However, a completely new figure suddenly appeared: that of the “savage.” Interestingly, Erichsen and Beck’s “savage” took his place in the textbook alongside invocations of European femininity. The relevant paragraph began by stating that the “higher man rises in the scale of civilisation,” the “more acute does his sensibility to pain appear to become, or possibly the less well able is he to bear it.” As a consequence, a

savage probably suffers less than a civilised man from any given injury, and hence may display more fortitude. An hysterical woman probably does not suffer more than one with a more healthy nervous system, but she complains more loudly, for she has her feelings in all things less under control. Race appears to exercise an influence in pain; some of the native races of India appear to suffer far less than Europeans under surgical operations of a similar kind (1884, 5–6 and 285–86. Also see 1888, 5–6 and 293).

In other words, Erichmen and Beck’s great Chain of Feeling positioned “civilised man” at one end and the “savage” at the other, but placed between these two extremes were “hysterical” women. The “savage” felt less pain, which enabled him to “display more fortitude.” It was a misleading “fortitude” because it was really simply due to physiological insensitivity. In contrast, the acute sensibilities of the “hysterical woman” were similar to the rest of her sex, but her lack of self-control meant that she possessed a lesser capacity for endurance.

Finally, the tenth edition of 1895 withdrew even token sympathy for the morally-“sordid” patient who was barely capable of bearing pain. While editions in the 1870s and 1880s warned against patients who had been “over-stimulated by habitual excesses in strong drinks, by which [they had] hoped to purchase temporary forgetfulness in the cares of a sordid life,” but in 1895, the last phrase was excised (1895, 5 and 303). Those who imbibed alcohol were possessed of weak, degenerate bodies, which were incapable of fortitude in times of suffering, and even poverty and deprivation could no longer be proffered as excuses.

## Complications due to “mental factors”

These debates about different people’s propensity to actually *feel* were complicated by the awareness that—irrespective of the “innate” sensibilities of a particular individual or group—emotional states dramatically affected levels of pain awareness and tolerance. This was what Erichsen was referring to when, in the 1860s, for the first time he included “trauma” as a factor in pain-sensation. Edward Deacon Girdlestone in the 1880s was also interested in the influence of “mental factors” on pain sensation. He recalled being informed of a butcher who had slipped while attempting to hook up a large piece of meat: the hook had penetrated his arm. “On being examined,” the butcher was described as

pale, almost pulseless, and expressed himself as suffering acute agony. The arm could not be moved without causing excessive pain; and in cutting off the sleeve he frequently cried out.

Yet, when the arm was exposed, it was discovered that the hook had simply pierced the sleeve of his coat. For Girdlestone, the message was clear. “The patient,” he explained,

was not a hysterical female, nor yet a poet; but *only a butcher*! Query: −- if a man’s imagination is able to *create* acute pain out of nothing, is it not reasonable to credit man with the power and the habit of *magnifying* already existing *little* pains? (1884, 22).

These “mental factors” were generally seen as arising out of particular environmental contexts. It became a cliché to observe that the “high excitement” of combat lessened the pain of being wounded. René Leriche’s exploration of surgical pain became an influential exposition about the way people in extreme situations might fail to register pain despite being severely wounded. In the context of the 1914–18 war, Leriche asserted that there was “all the difference in the world between the reactions [to wounding and surgery] of a European and those of an Asiatic or an African.” He was profoundly impressed by the “almost complete indifference to pain” shown by Russian allies and claimed that his Russian colleagues advised him that it was “useless to give an anæsthetic to certain Cossacks before operating on them—because… they felt nothing,” Leriche decided to experiment. One day, he

disarticulated, without any anæsthetic, though with considerable repugnance on my part, three fingers and their metacarpals of one wounded Russian, and the whole foot of his comrade. Neither one man nor the other showed the least tremor, but turned the hand or raised the leg when asked to do so, and not showing even the slightest sign of momentary weakness, just as if under the most perfect local anæsthetic.

Leriche was not making an argument about the propensity of various nationalities or “races” to ignore pain. In attempting to explain this strange phenomenon, Leriche turned neither to the racial sciences nor to other ideas about innate physiological differences: rather, he insisted, a “mental factor” had to be acknowledged. He noted that Russian soldiers possessed the same physiology (or, to use his mannered language, the same “appropriate apparatus”) as other people. Consequently, a psychological dimension must have intervened by either suppressing the expression of pain or diminishing its acuteness:

We all know that, in certain circumstances, we do not suffer pain, when we ought, in fact, to be acutely conscious of it. Many wounded men, in the heat of action, have had their flesh lacerated and torn, without being conscious of anything. When our attention is intensely fixed on something, we may be quite unconscious of pain, and may be prevented from feeling, as we otherwise would, the lacerations of our nerve endings and of our nerves.

Willpower had “certainly nothing to do with it.” Rather, the explanation had to be in “certain movements of our hormones, or of the blood,” which were “diverted into directions other than normal, as the result of fixed attention or of emotion and have the effect of displacing the area (or altering the atmosphere) of pain.” In addition, he noted, the appreciation of pain was affected by

diet, vitamins, atmospheric conditions, and everything that is capable of bringing conditional reflexes into action; for certainly the mechanism of sensibility cannot escape the effects of association which are produced in us by actions regularly repeated (1938, 2–3 and 7).

Leriche’s observations, which were drawn from his experiences during the World War I, were perceptive but anecdotal. They were confirmed by a more systematic study based on the second world war of the twentieth century. Lt. Col. Henry K. Beecher, who served in combat zones on the Venafro and Cassino fronts, was struck by the fact that many severely wounded men did not complain of pain. Medical officers found that there was no necessary correlation between the size and depth of any specific wound and men’s expressions of suffering. Rather than anecdote, Beecher decided to explore this paradox systematically, questioning 215 seriously wounded men. To his surprise, three-quarters did not report experiencing significant pain. One third claimed to be feeling no pain at all, while another quarter said they were experiencing only slight pain. Of course, there were differences related to particular sites of wounding. Penetrating abdominal wounds, for instance, were more painful (nearly half of men with such wounds admitted that their pain was “bad”) than penetrating wounds of the thorax (12 %) or cerebral wounds (7 %). Remarkably, three quarters of all seriously wounded men did not even ask for pain relief, despite the fact that being asked the question would have served as a reminder that relief was available.

What was happening? It was relatively easy to explain the severity of suffering for men with abdominal wounds: such wounds caused blood and “intestinal contents” to spill into the peritoneal cavity, spreading infection. However, even significant numbers of these men did not complain of serious pain. Perhaps, Beecher speculated, men who had been wounded were simply less sensitive generally. But this explanation failed to account for the fact that “a badly wounded patient who says he is having no wound pain will protest as vigorously as a normal individual at an inept venipuncture.” Instead, Beecher argued, there must be a difference between wounds caused in civilian contexts (a car accident, for example) and those caused during combat. Perhaps the strong emotions aroused in combat were responsible for the absence of acute pain. Pain might also be alleviated by the fact that wartime wounding would release a soldier

from an exceedingly dangerous environment, one filled with fatigue, discomfort, anxiety, fear and real danger of death, and gives him a ticket to the safely of the hospital. His troubles are about over, or he thinks they are.

This was in contrast to civilian accidents, which only heralded in “the beginning of disaster” (1946, 96–105).

Beecher’s findings were profoundly influential in post-war reworking of notions of pain. As pain researchers Harold Wolff and Stewart Wolf found in the 1950s, most people perceived pain at around similar intensities, but their threshold for reaction varied widely. This did not surprise them since the “ability to perceive pain depends upon the intactness of relatively simple and primitive nerve connections.” while reacting to pain was “modified by the highest cognitive functions and depends in part upon what the sensation *means* to the individual in the light of his past experiences” (1958, 19 and 22). It was a classic statement that became the dominant way of thinking about pain in the second half of the twentieth century.

This distinction between perceiving and reacting to pain received a significant boost from 1943 when physicians began operating on the brains of people suffering agonizing and intractable pain. To everyone’s astonishment, lobotomies (and its numerous surgical variations, such as prefrontal leucotomies and topectomies) had an unexpected effect: after the operation, patients were still aware that they were experiencing something they identified as pain but were utterly undisturbed by it (Ostenasek 1948, 229). As leading psychosurgeons Walter Freeman and James W. Watts wrote in their influential textbook *Psychosurgery. In the Treatment of Mental Disorders and Intractable Pain* (1950),

The individual might use the same terms to describe the pain after the operations as he used before. [But] the attitude was different. Fear seemed to have gone. The pain was present, but it was a sensation, rather than a threat (353).

These patients could still perceive when they were being pricked with pins, scratched, or subjected to extremes of heat or cold, and their threshold for identifying the level of stimulation was unaffected: they simply were not emotionally affected by it.

Physiologists, psychologists, and sociologists enthusiastically sought to confirm this distinction between perception and reaction, instigating a huge number of experiments seeking to document the two very distinctive thresholds. Like their predecessors, they shared a curiosity about “racial” and ethnic difference, contrasting (for instance) the pain thresholds of northern and southern Europeans, Mi’kmaq Indians, Native Alaskan Indians, Eskimos, or African-Americans (For instance, see Chapman and Jones 1944; Jewsbury 1951, 336; Sherman 943, 441; Meehan, Stoll, and Hardy 1954, 397–400; Zborowski 1969; Zborowski 1958, 256–68). Some of the most interesting of these experiments involved testing the effect of group cohesion and identification on pain tolerance. For instance, in the late-1950s researchers at McGill University set out to manipulate “an ethnocentric prestige motive” or inter-group rivalry. They discovered that they could change the level at which people tolerated pain. For instance, when Jewish women were casually informed that, compared to non-Jews, Jews were “inferior” in their capacity to withstand pain, the Jewish women’s tolerance levels soared. This result was not replicated when Protestant women were told the same thing about non-Protestants generally. However, when Protestant women were told that Christians tolerated less pain than Jews, the pain tolerance level of these Protestant women increased. By specifying the rivalrous comparison group (Jews) as opposed to the vague “non-Protestant” category, the researchers increased the salience of these women’s identification. In other words, pride in one’s group identification (as Jews for the Jewish women and as Christians as opposed to Jews for the Protestant women) made these women willing to endure discomfort for the sake of their group’s reputation. For all participants, the ability to tolerate pain was assumed to confer a high status of their group (Lambert, Libman, and Poser 1960, 350–57).

Research into the way the acuteness, salience, duration, and affective qualities of pain varied according to the meanings attached to the noxious stimuli bolstered the arguments of many scholars that “the pain of the laboratory” and “the pain-malady” could not be considered “as one and the same thing,” according to Réne Leriche in 1938. Pain was more than tissue damage. It was intrinsically affected by interactions with other people and the environment. Experimental pain was “the result of a short excitation repeated from time to time,” Leriche observed: in contrast, the pain people experiencing in their everyday lives were encountering “a continuous phenomenon, with special paroxysms certainly, but with a background which remains unchanged over months or even years.” This was a very different experience to the “transient disagreeable sensation of pricking or pinching that can be provoked in a healthy individual.” He castigated physiologists for adhering to a concept of pain that was “too mechanical, too purely artificial, to be capable of reconciliation with which we doctors see in the human patient,” and pleaded with them to pay attention to “the affective or mental quality” of pathological pain syndromes (481–83).

Not surprisingly, that other great pain-researcher—Beecher—agreed with Leriche. He opposed studies that sought to understand the mechanisms of pain in experimental settings, artificially producing pain through pricking people’s skin, giving them electric shocks, applying heat to their foreheads or teeth, or tightening a tourniquet around their limbs. In “Experimental Pharmacology and Measurement of the Subjective Response” (1952), Beecher maintained that “no one who has worked with problems of pathological pain can doubt the importance in their field of the environment, of emotional factors, or the reaction to pain.” It “requires little imagination,” he continued,

to suppose that the sickbed of the patient in pain, with its ominous threat against his happiness, his security, his very life, provides an entirely different milieu (*and reaction*) than the laboratory, with its dispassionate and unemotional atmosphere.

In other words, pain experiences consist of both perceptive aspects and reactive ones, and the only way to truly understand human suffering was through observing and listening to the “man himself in real pain of pathological origin” (159–60).

These debates reached their height in 1965 when Ronald Melzack and Patrick Wall effectively overturned commonly-understand mechanism of pain with their Gate Control Theory, introducing the idea of a “gating mechanism” in the dorsal horns of the spinal cord that allowed the perception of pain to be modified. Crucially, the Gate Control Theory insisted that sensory, cognitive, and affective processes influence people’s experience of pain. In effect, mind and the body became fully integrated (971–79).

## Conclusion

This article began with the reminiscences of E.M.P. in 1896. He worked in the surgical-dressing room of a hospital in an area of London with a large immigrant population, but his dismissal of the pain of fellow-humans is alive and well in clinical settings today. It was not until the 1980s that the under-treatment for pain in minority groups and women began to be addressed, and even today these patients continue to be strongly affected by prejudices about their high threshold for perceiving pain (Green, Anderson, Baker, et al. 2003, 277–94 and Hoffman and Tarzian 2001, 13–27). Debates about the relative sensitivity of different people to noxious stimuli were not merely academic. The seriousness of people’s sufferings was calibrated according to such characterisations and sympathy was unevenly rationed. Myths about the lower susceptibility of certain patients to painful stimuli justified physicians and other care-givers prescribing fewer and less effective analgesics and anaesthetics, which affected all the groups discussed here. The belief that not every person-in-pain suffered to the same degree was systemic to hierarchical systems generally, shifting in line with other social changes—including slave emancipation, anti-imperialism, unionism, and suffrage. Indeed, the process of labelling was itself indicative of power. After all, it was the colonialist’s voice declaring that indigenous peoples were insensible to pain; the slave-owner professing the extraordinary hardiness of Africans; the professor informing us that the miner’s back was sturdy enough to bear the weight of coal; and the male anthropologist failing to recognise suffering in the birthing-hut in Kenya while his medical colleague in London recognised the exquisite sensitivities of European women when their infant’s head emerged “through the maternal parts like a veritable stone” (Knaggs 1931, 6 and 9). In each case, commentators retained contradictory ideas simultaneously: the humble status of workers and immigrants meant that they were likely to be insensitive to noxious stimuli; the profound inferiority of these same patients meant that they were especially likely to respond with “exaggerated” sensitivity. Pain-assignation claimed to be based on natural hierarchical schemas, but the great Chain of Feeling was more fluid than it seemed. The question of “whose pain is heard” was not only correlated with power differentials between different groups in society (in which case, the solution was to improve access to resources); patients considered to be “truly” in pain were also directly constituted by those differentials.
